# Analysis of the Effectiveness of Decontamination Fluids on the Level of Biological Contamination of Firefighter Suits

**DOI:** 10.3390/ijerph17082815

**Published:** 2020-04-19

**Authors:** Andrzej Polanczyk, Aleksandra Piechota-Polanczyk, Anna Dmochowska, Malgorzata Majder-Lopatka, Zdzislaw Salamonowicz

**Affiliations:** 1 The Main School of Fire Service, Slowackiego 52/54 Street, 01-629 Warsaw, Poland; admochowska@sgsp.edu.pl (A.D.); mmajder@sgsp.edu.pl (M.M.-L.); zsalamonowicz@sgsp.edu.pl (Z.S.); 2 Department of Medical Biotechnology, Jagiellonian University, Gronostajowa 7 Street, 30-387 Krakow, Poland; aleksandra.piechota-polanczyk@uj.edu.pl

**Keywords:** biological decontamination, Gram’s method, decontamination, firefighter special outfit

## Abstract

The scope of tasks of chemical and ecological rescue procedures includes prevention of terrorist attacks with biological weapons. After each action, firefighters are obliged to clean and disinfect their outfits to prevent the potential spreading of harmful microorganisms. This study aimed to analyze the effectiveness of decontamination fluids used to disinfect firefighter’s suits. Two types of clothes were analyzed: special combat clothing (NOMEX), and the heavy gas-tight chemical type 1a suit. Swabbed places were cut out and sterilized mechanically using detergent and alcohol. Each time, smears were made on sterile glass, fixed in pure ethanol and stained using the Gram method. After this, the staining samples were air dried and photographed under a light microscope at magnification 1000×. Each smear was made in triplicate and the relative number of stained microorganisms was analyzed using ImageJ software. The results showed that detergent significantly decreased the number of pathogens in the chest area on the NOMEX suit and the type 1a-gas-tight clothing and was more effective than alcohol, especially in case of the NOMEX suit. In conclusion, the detergent was more efficient in decontaminating the NOMEX outfit than the heavy gas-tight clothing, whose surface was better cleaned by the alcohol.

## 1. Introduction

Without knowing the causes of disease formation, the development of methods for biological contamination treatment is impossible [1,2,3]. The presence of biological threats does not only refer to war conflicts, bioterrorism, and the criminal world, but also to chemical spills and biological contamination [4,5,6]. One of the major problems associated with chemical warfare is the use of proper protective clothing [7,8,9]. Numerous occupations require the wearing of personal protective equipment [10,11] such as protective hoods, coats, helmets, and self-contained breathing apparatus wear by firefighters [12]. According to the Polish regulation of the Minister of Health from 25 April 2005, “on harmful biological factors for health in the work environment and health protection of workers exposed to these factors”, employees in some enterprises, institutions, and companies are also exposed. These include, but are not limited to, work related to (1) food production; (2) cultivation of agriculture, as well as places where there is contact with products of animal origin or animals; (3) waste management and wastewater treatment; (4) health care and diagnostic, clinical, or veterinary laboratories.

Pursuant to the ordinance of the Minister of Interior and Administration on the detailed organization of the national rescue and fire-fighting system, the State Fire Service includes chemical and ecological rescue, whose purpose is to plan, organize, and carry out rescue operations in such a way as to remove or reduce the threat caused by dangerous substances. The scope of tasks of chemical and ecological rescue also includes the prevention of terrorist events with the use of biological factors including bacteria (Regulation of the Minister of Interior and Administration of 3 July 2017).

Pathogens, among which are bacteria, can be spread by air-droplet, air-dust, food, by the bite of blood-born arthropods, and damaged skin or through carriers [13,14,15]. Firefighting is one of the most challenging and dangerous professions [16,17]. During action, the firefighter’s skin is exposed to contamination in between interface regions of the turnout jacket and trousers, or through the cross-transfer of contamination on gear to skin [18]. Additionally, firefighters are at increased health risks of simultaneous exposure to multiple chemicals [19]. Biological pathogens cause severe or fatal diseases. However, not all are infectious, but to determine this, it is necessary to take a sample and examine it in laboratory conditions. Analytical methods can determine the presence and type of bacteria that caused the disease. Therefore, in most cases, it is suitable enough to identify the shape of the bacteria under a microscope. In other cases, dyeing methods, such as Gram staining, can be applied. This method helps to differentiate bacteria and assign them to a specific group, which may help to choose the proper way of decontamination to minimize the risk of further spreading pathogens.

The process of decontamination is defined as “a set of actions carried out on the spot by emergency services, in order to reduce the absorption of substances by the injured, stop the spread of contamination and prevent secondary contamination of rescuers” (Regulation of the Minister of Interior and Administration of 3 July 2017). The decontamination process can be carried out in four ways: (1) mechanical, (2) chemical, (3) physical, and (4) mixed. The decontamination options include wet decontamination, air decontamination, and dry decontamination [20]. The decontamination process consists of immersing or rubbing with absorbent materials of a contaminated person or equipment. To this end, appropriate substances should be selected that are adjusted to the threat. In the case of biological hazards, only cleaning and disinfecting agents can be used for decontamination purposes [21]. Types of preparations are classified by the State Sanitary Inspection of the Ministry of the Interior depending on the microorganism found. These agents are fungicidal, tuberculocidal, bactericidal, virucidal, and sporicidal. The most commonly used preparations in the fire service are: PearSafe, Virkon, BX 29, and BX 24. However, soap and alcoholic solutions are also used. For instance, surfactants such as dish soap are designed to surround lipid molecules and liberate them from surfaces so that water can then take them away [20]. Therefore, the aim of the study was to analyze the effectiveness of the most commonly used decontamination fluids on biological contamination level of a firefighter’s suit.

This paper is organized as follow. In Section 2, the analyzed material, investigated method and statistical analysis applied in the paper are described. Section 3 presents the results of the Gram staining of firefighters’ suits. In Section 4, obtained results are discussed, while Section 5 concludes the paper.

## 2. Materials and Methods

### 2.1. Sample Collection

**Figure 1:**
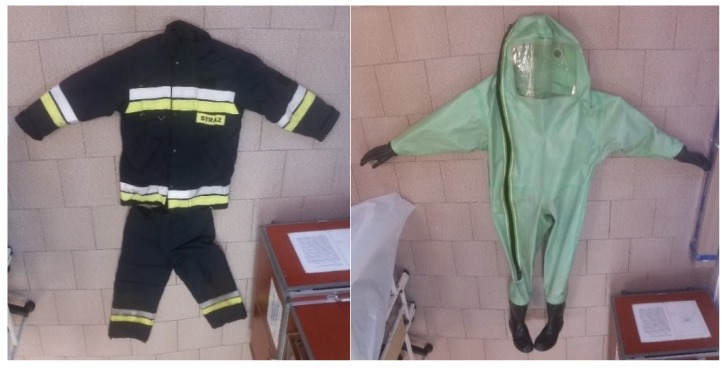
Special combat clothing (NOMEX clothing) (on the **left**) and type 1a heavy gas-tight clothing (on the **right** side).

Two types of clothes were analyzed during the tests: special combat clothing (NOMEX), which is most often used during rescue operations threatened with biological contamination, and the heavy gas-tight chemical type 1a suit (Figure 1). Four suits of each type were analyzed. The outfits were used for one month in field conditions (during cadets’ one-month training in closed campus) before being tested for biological contamination using Gram staining.

Special combat clothing (NOMEX) consists of four layers: (1) outer fabric (outer layer), (2) waterproof and vapor permeable membrane, (3) thermal insulation layer, and (4) lining. The outer fabric consists of 60% aramid fibers, while the other 40% is viscose. The membrane contains 50% polyester and 50% polyurethane. The only component of the thermal insulation insert is the aramid fiber. The lining was made of 50% aramid fibers and 50% viscose.

Meanwhile, the type 1a gas-tight clothing consists of polyamide covered with a polyvinyl chloride (PVC) layer on both sides. Similar material is used to make shoes and a visor. The gloves are made of a mixture of nitrile rubber and chloroprene rubber. These two suits are the most commonly used during events with biological threats, therefore they were chosen for the analysis.

**Figure 2:**
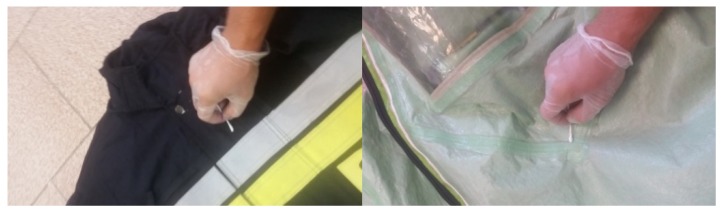
An example of sample collection from special NOMEX clothing (on the **left**) and type 1a heavy gas-tight clothing (on the **right** side).

Sterile cotton swabs were used to take biological samples from 3 cm^2^ areas of analyzed suits (Figure 2). Each time, 3 separate swabs were taken. Tested places included the chest, the front of the thigh, and hard to reach places, such as the knee and elbow flexion, armpit, and crotch. Swabs were transferred each time on sterile glass to create smears which were further fixed in pure ethanol.

**Figure 3:**
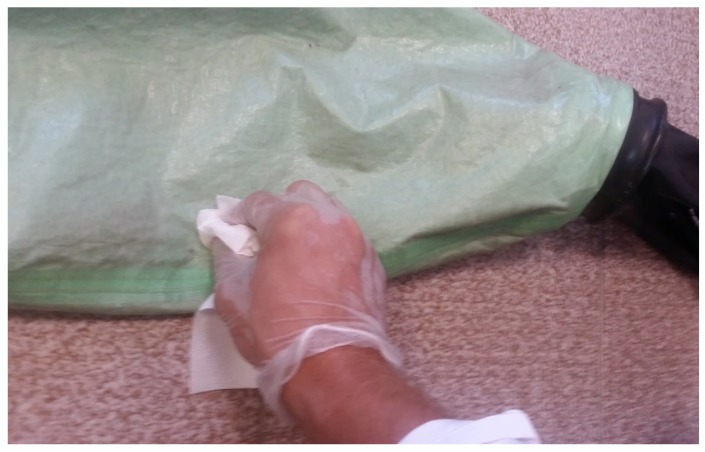
An example of the decontamination of the type 1a heavy gas-tight clothing.

Next, swabbed places were sterilized mechanically by rubbing using tap water for 20 s with 20% soap or 98% alcohol (Figure 3). When the fabric was dry, (which took around 10 min for detergent and 1 min for alcohol) swabs from the same areas were collected, transferred onto sterile glass and fixed with ethanol. Each time, the samples were collected in triplicate and subjected to Gram staining.

### 2.2. Gram Staining

To visualize the bacteria and distinguish Gram-positive and Gram-negative bacterial species, the previously described Gram staining method was used [22]. In this method, the samples are briefly stained with crystal violet, which joins peptidoglycans in the bacterial wall, before adding iodine, which creates complexes with crystal violet, then de-staining with alcohol, which washes out the crystal violet–iodine complex from Gram-negative bacteria. Finally, the samples are stained with fuchsin, which visualizes Gram-negative bacteria. After staining, samples were air dried and photographed under a light microscope at magnification 1000×. After staining, Gram-positive bacteria should be observable as purple-blue and Gram-negative bacteria should be pink-red.

Quantification of the bacteria was performed using ImageJ software with the “analyze particles” tool after brightness/contrast adjustment, described in detail in [22].

### 2.3. Statistical Analysis

Data are presented as mean ± standard error (SEM). Comparisons between two types of suits and decontamination methods was calculated using one-way ANOVA with Bonferroni’s post-test. Comparison between two groups was performed using the unpaired Student’s *t*-test for normally distributed variables or non-parametric Mann–Whitney U-test for non-normally distributed variables after verification of normality. Outliers were calculated with Grubbs’ test (Statistica 12.0 software). Data were considered statistically different when *p* < 0.05.

## 3. Results

**Figure 4:**
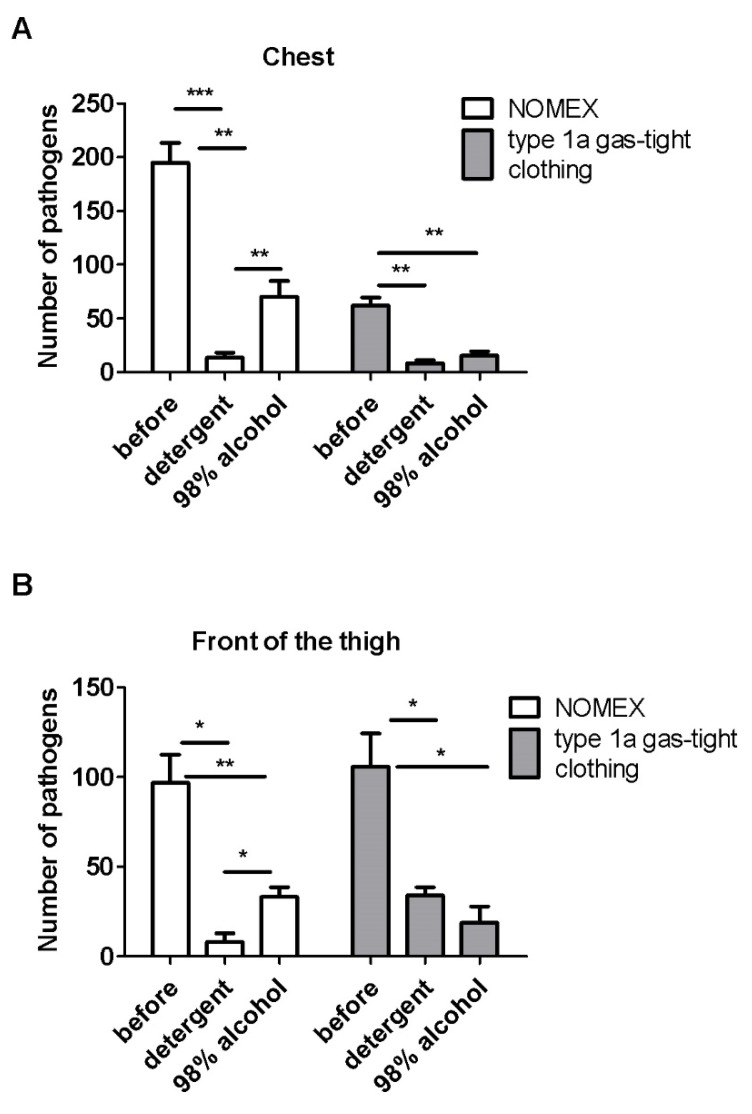
The effectiveness of decontamination of NOMEX and type 1a gas-tight clothing, measured as the number of pathogens on the chest (**A**) and front of the thigh (**B**). Mean ± standard error (SEM). N = four suits per group and three swabs from each place. * *p <* 0.05, ** *p* < 0.01, *** *p* < 0.001 calculated with Students *t*-test.

The results indicated that the decontamination process effectively decreased the number of pathogens on each analyzed area. Figure 1 presents changes in pathogens number from “easy to reach” places such as the chest and front of the thigh on NOMEX suits and type 1a gas-tight clothing. The detergent significantly decreased the number of pathogens in the chest area on the NOMEX suit (*p* < 0.001) and the type 1a gas-tight clothing (*p* < 0.01) and was more effective than alcohol, especially in case of the NOMEX suit (*p* < 0.01) (Figure 4A). Similar results were gathered for the NOMEX suit on the front of the thigh, but not for the type 1a gas-tight clothing, where both reagents were similarly effective. (Figure 4B).

The effectiveness of the decontamination with the detergent for the chest was equal to 92.9% and 86.3% for the NOMEX suit and the type 1a gas-tight clothing, respectively. For alcohol, it was equal to 63.9% and 74.8% for the NOMEX suit and the type 1a gas-tight clothing, respectively. In the case of the front of the thigh, the detergent disposed of 91.6% and 67.9% of pathogens from the NOMEX suit and the type 1a gas-tight clothing, respectively. Alcohol removed 65.6% and 82.2% for the NOMEX suit and the type 1a gas-tight clothing.

**Figure 5:**
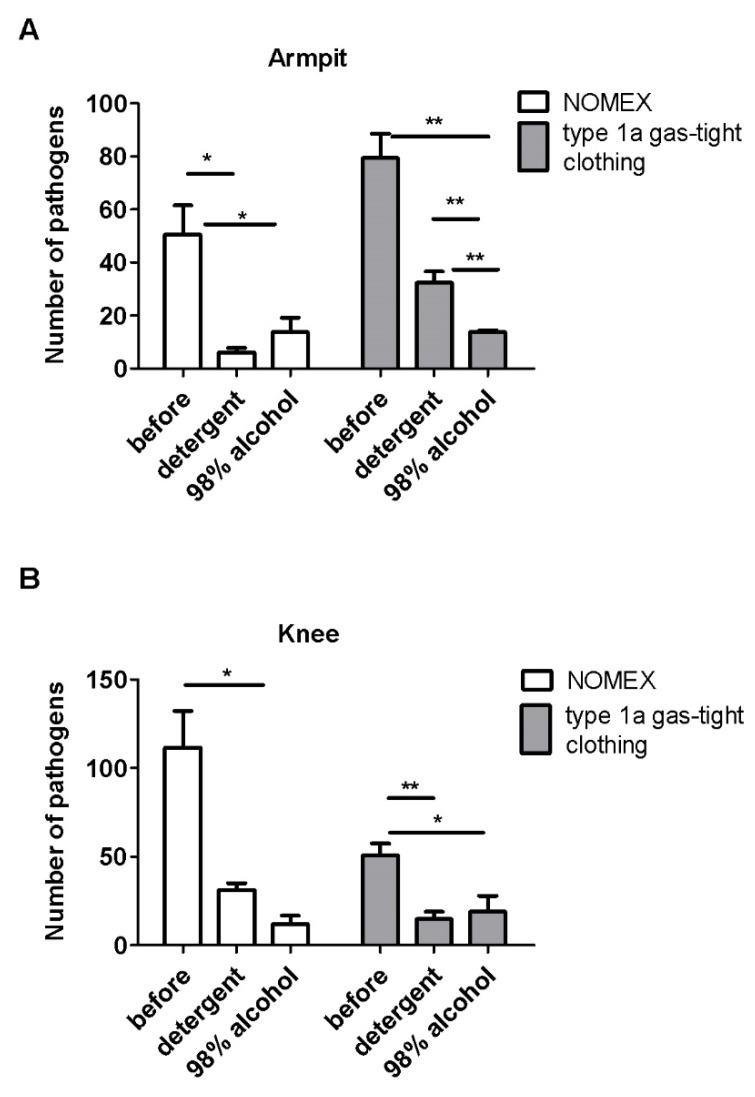
The effectiveness of decontamination of NOMEX and type 1a gas-tight clothing, measured as number of pathogens on the hard to reach areas such as the armpit (**A**) and knee (**B**). N = four suits per group and three swabs from each place. Mean ± SEM. * *p* < 0.05, ** *p* < 0.01 calculated with Students *t*-test.

**Figure 6:**
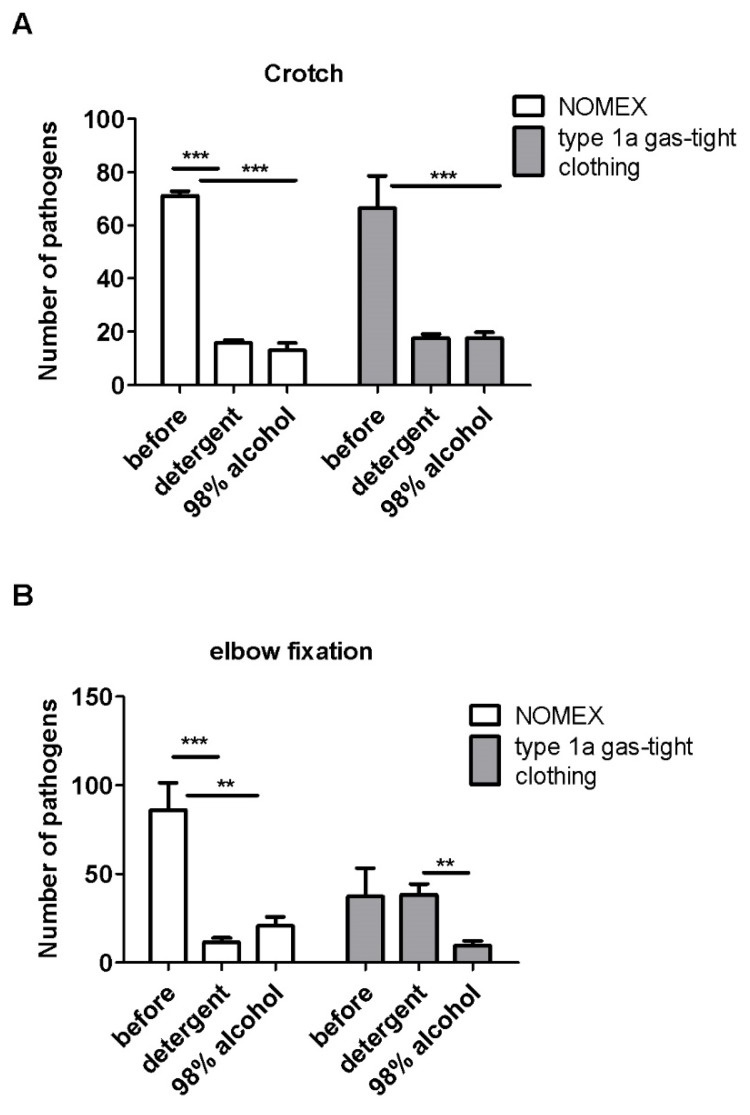
The effectiveness of decontamination of NOMEX and type 1a gas-tight clothing, measured as number of pathogens on the hard to reach areas such as crotch (**A**) and elbow fixation (**B**). N = 4 suits per group and 3 swabs from each place. Mean ± SEM. ** *p* < 0.01, *** *p* < 0.001 calculated with Students *t*-test for normally distributed data and U-Mann Whitney test for non-normally distributed variables.

Analysis of effectiveness of the decontamination of the hard-accessible areas such as the armpit, knee, crotch, and elbow fixation showed that, in most cases, the detergent was more effective in decreasing the number of pathogens from the NOMEX suit compared to the type 1a gas-tight outfit (Figure 5 and Figure 6).

The decontamination effectiveness on the armpit (Figure 5A) and knee (Figure 5B) area showed that both decontamination fluids significantly reduced the number of pathogens on the NOMEX and type 1a gas-tight clothing. On the armpit, we also noticed that alcohol was more efficient than detergent in decreasing the number of pathogens on the type 1a gas-tight clothing (*p* < 0.01), and similarly effective in other analyzed cases.

The effectiveness of the decontamination with the detergent for the armpit was equal to 80.8% and 59.2% for the NOMEX suit and the type 1a gas-tight clothing, respectively. For alcohol, it was equal to 87.4% and 80.5% for the NOMEX suit and the type 1a gas-tight clothing, respectively. Additionally, the detergent removed 71.9% and 70.8% from the knee for the NOMEX suit and the type 1a gas-tight clothing, respectively. Alcohol discarded 89.3% and 62.9% for the NOMEX suit and the type 1a gas-tight clothing, respectively.

A significant decrease in the number of pathogens was observed for the crotch (Figure 5A, *p* < 0.001) and elbow fixation (Figure 6B, *p* < 0.01 for alcohol and *p* < 0.001 for detergent) for the NOMEX suit. On the other hand, alcohol—but not detergent—was efficient in cleaning the crotch (Figure 5B, *p* < 0.001) on the type 1a gas-tight clothing. Both detergents were ineffective in the decontamination of the elbow fixation on the type 1a gas-tight suit (Figure 6B).

Furthermore, the effectiveness of the decontamination with the detergent for the crotch was similar and equal to 77.6% and 73.5% for the NOMEX suit and the type 1a gas-tight clothing, respectively. Alcohol was slightly more effective on the NOMEX suit (81.6% vs. 73.5% for the NOMEX suit and the type 1a gas-tight clothing, respectively). Additionally, the detergent removed 91.6% of pathogens from the elbow fixation on the NOMEX suit, but only 67.9% of pathogens from the type 1a gas-tight clothing. The opposite observation was made for alcohol, which discarded 65.6% of pathogens from the NOMEX suit and 82.2% of pathogens from the type 1a gas-tight clothing.

## 4. Discussion

Firefighters, police, and/or military officers are often required to wear personal protective suits during emergency actions [19,22]. The characteristics of firefighting work involves exhibition on the chemical or biological factors [10,12] which is limited by personal protective equipment, e.g., turnout jackets, trousers, hoods, and gloves [16]. Additionally, Costello et al. showed that explosive ordinance disposal is often required to wear additional clothing that repels the contact of chemical or biological agents from the skin [10]. Due to numerous clothing types used by firefighters, different methods of cleaning, including laundering, should be regularly applied. According to some papers, this should be conducted more often than once or twice per year [20], or before being reused [23]. Interestingly, a study conducted by Stull et al. determined that the laundering procedure affects the ability of the gear to resist water penetration for a non-breathable moisture barrier material [21]. In our work, we proved that high risk occupations are associated with the appearance of bacteria on the surface of combat suits, which can be further cleaned with selected detergents.

Different disinfection substances are applied for the cleaning process of protective suits, for instance, Kenneth et al. successfully decontaminated firefighters’ suits with isopropanol [20]. In our work, we used ethanol and detergent and showed that both disinfectant factors were effective in removing biological contamination, however, to different extents. The studies of others indicated that the effectiveness of the mixture of water and dish soap on turnout jackets and pants depends on the detergent and surface type, and the application of 70% isopropanol removes less than 40% of contamination form non-porous surfaces, while the application of benzalkonium chloride, a surfactant, is more effective on non-porous surfaces [20].

Moreover, we showed that image processing methods may be applied for the recognition of microorganisms on different type of materials. In this work, image analysis was applied for the quantitative analysis of protective suits before and after decontamination. A similar approach was used by Czernicka et al., who previously described the bioactivity assessment of crude extracts with the help of a freeware ImageJ software [24]. Similarly, Bera et al. estimated the bacterial concentration in fibrous substrates through a combination of scanning electron microscopy and ImageJ software [25].

### Limitation to the Study

In this work, two types of firefighters’ suits, which are the most commonly used during actions, were analyzed and treated with two easily accessible decontamination fluids, e.g., detergent and alcohol. Instead of the recognition of particular bacteria, the number of bacteria before and after the decontamination process was analyzed. In the future work, we plan to extend the analyzed group of protective suits and detergents.

## 5. Conclusions

The aim of the study was to analyze the effectiveness of decontamination fluids on the biological contamination of firefighter suits. The results indicated that none of the two tested decontamination fluids completely removed pathogens from the selected areas. In the case of NOMEX clothing, detergent proved to be more effective than alcohol. The detergent, due to the substances contained in it as well as used friction, produced active foam which effectively reduced the number of pathogens on the analyzed areas. The foam allowed us to interfere in the deeper layers of the material, bringing the bacteria to the surface and removing them. For the type 1a heavy gas-tight clothing, alcohol was effective, because it has antibacterial properties that enabled the removal of microbes from the PVC surface.
